# Machine Learning-Based Approach to Predict Intrauterine Growth Restriction

**DOI:** 10.7759/cureus.41448

**Published:** 2023-07-06

**Authors:** Elham Taeidi, Amene Ranjbar, Farideh Montazeri, Vahid Mehrnoush, Fatemeh Darsareh

**Affiliations:** 1 Mother and Child Welfare Research Center, Hormozgan University of Medical Sciences, Bandar Abbas, IRN; 2 Fertility and Infertility Research Center, Hormozgan University of Medical Sciences, Bandar Abbas, IRN

**Keywords:** prenatal maternal screening, intrauterine growth restriction (iugr), decision tree classification, gradient boost algorithm, random forest, artificial intelligence, deep learning, machine learning, fetal growth restriction, intrauterine growth restriction

## Abstract

Introduction: Creating a prediction model incorporating multiple risk factors for intrauterine growth restriction is vital. The current study employed a machine learning model to predict intrauterine growth restriction.

Methods: This cross-sectional study was carried out in a tertiary hospital in Bandar Abbas, Iran, from January 2020 to January 2022. Women with singleton pregnancies above the gestational age of 24 weeks who gave birth during the study period were included. Exclusion criteria included multiple pregnancies and fetal anomalies. Four statistical learning algorithms were used to build a predictive model: (1) Decision Tree Classification, (2) Random Forest Classification, (3) Deep Learning, and (4) the Gradient Boost Algorithm. The candidate predictors of intrauterine growth restriction for all models were chosen based on expert opinion and prior observational cohorts. To investigate the performance of each algorithm, some parameters, including the area under the receiver operating characteristic curve (AUROC), accuracy, precision, and sensitivity, were assessed.

Results: Of 8683 women who gave birth during the study period, 712 were recorded as having intrauterine growth restriction, with a frequency of 8.19%. Comparing the performance parameters of different machine learning algorithms showed that among all four machine learning models, Deep Learning had the greatest performance to predict intrauterine growth restriction with an AUROC of 0.91 (95% confidence interval, 0.85-0.97). The importance of the variables revealed that drug addiction, previous history of intrauterine growth restriction, chronic hypertension, preeclampsia, maternal anemia, and COVID-19 were weighted factors in predicting intrauterine growth restriction.

Conclusions: A machine learning model can be used to predict intrauterine growth restriction. The Deep Learning model is an accurate algorithm for predicting intrauterine growth restriction.

## Introduction

Intrauterine growth restriction is a common and complicated obstetric problem. Intrauterine growth restriction is the fetus's inability to reach its full growth potential due to fetoplacental factors and maternal factors, most commonly placental dysfunction [1]. Based on the commonly used definition of intrauterine growth restriction as a fetus weight below the 10th percentile, the incidence of intrauterine growth restriction has been estimated to be about 10%-15% [2]. Intrauterine growth restriction is one of the main causes of neonatal mortality and morbidity, with significant consequences in fetal, neonatal, and adult life [3]. Constant progress in clinical practice, particularly in the definitions, diagnosis, and management of intrauterine growth restriction, necessitates efforts to effectively translate these changes to a diverse range of midwifery care providers [4]. Early detection of intrauterine growth restriction allows for the selection of patients for follow-up, allowing for closer monitoring and better clinical management. Because they allow for the interpretation of model parameters, clinical risk prediction models such as Logistic Regression are commonly found in medical domains. However, machine learning can learn data patterns in higher dimensions.

Despite the high prevalence of intrauterine growth restriction and its potentially severe consequences for patients, there has been little research into identifying its predictors. Some studies concentrate on individual factors that may lead to health problems in children born with this condition later in life. Because of its high potential for using complex mathematical operations to compute large amounts of data, machine learning techniques for clinical data analysis are becoming increasingly popular. It may aid health researchers in analyzing and visualizing large amounts of complex data and processing results quickly and accurately. As a result, creating a single customized algorithm based on the comparison and evaluation of various techniques can be a proper solution for the current application [5,6]. The application of machine learning has dramatically evolved in the medical field over the past decade [5]. However, in the diagnosis of intrauterine growth restriction, the evidence is limited. According to a meta-analysis of 10 studies conducted to predict the role of machine learning in predicting intrauterine growth restriction, machine learning techniques were found to be effective in predicting and identifying fetuses at risk for intrauterine growth restriction during pregnancy, with a pooled overall diagnostic performance of sensitivity = 0.84, specificity = 0.87, positive predictive value of 0.78, negative predictive value of 0.91, and a diagnostic odds ratio of 30.97. Among different machine learning models, the Random Forest-Support Vector Machine model with 97% accuracy showed the best diagnostic performance in predicting intrauterine growth restriction [7]. However, we believe more research in this field is needed to reach a better conclusion. Therefore, this study aims to evaluate the performance of machine learning-based models to predict intrauterine growth restriction.

## Materials and methods

This cross-sectional study was carried out at Khaleej-e-Fars Hospital, a tertiary hospital in Bandar Abbas, Iran, which has a birth rate of 400-5000 babies annually. The period of study was from January 2020 to January 2022. Women with singleton pregnancies above the gestational age of 24 weeks who gave birth during the study period were included. Intrauterine growth restriction was confirmed on ultrasound when the fetal abdominal circumference was less than two standard deviations from the mean value [1]. Exclusion criteria included multiple pregnancies and fetal anomalies.

The candidate predictors of intrauterine growth restriction for all models were chosen based on expert opinion and prior observational cohorts. Predictor factors included maternal age, maternal education, living place, inadequate prenatal care (defined by the researcher as less than three prenatal care visits), drug addiction, anemia, cardiovascular disease, chronic hypertension, pregnancy-induced hypertension, pyelonephritis, hepatitis, COVID-19, overt diabetes, gestational diabetes, thyroid dysfunction, parity, preeclampsia, and previous history of intrauterine growth restriction. These were obtained from the patients' medical records. Four statistical learning algorithms were used to build a predictive model: (1) Decision Tree Classification, (2) Random Forest Classification, (3) Deep Learning, and (4) the Gradient Boost Algorithm. Python was chosen as the programming language for creating the machine learning model. The machine learning algorithm was implemented by the Python machine learning library. It includes a comprehensive set of cutting-edge machine-learning algorithms [8].

Internal validation was performed using K-fold cross-validation. Using a random number generator, the 8,683 cases were randomly assigned to either the "training set" (70%) or the "test set" (30%). The rate of intrauterine growth restriction and non-intrauterine growth restriction groups in the training and test sets was kept constant with the original dataset. We arranged the parameters of the prediction models using the training set and evaluated their performance using the test set. By repeating these 10 times, the average performance was calculated.

To assess the performance parameters of the algorithms, metrics such as the area under the receiver operating characteristic curve (AUROC), accuracy, precision, and sensitivity were measured in the test set. Because AUROC is a widely used index to describe a machine learning model's ability to predict outcomes, we used it as the primary performance metric. The metrics ranged from zero to one, with values closer to one showing greater performance. The following equation was used to assess each metric [9].

For a clinical risk prediction model, the AUROC tells us the probability that a randomly selected patient who experienced a particular event will have a higher predicted risk score than a randomly selected patient who did not experience an event. Accuracy is defined as (correctly predicted as non-intrauterine growth restriction cases) + (correctly predicted as intrauterine growth restriction cases) / total case. Sensitivity is defined as (correctly predicted as intrauterine growth restriction cases) / (correctly predicted as intrauterine growth restriction cases + incorrectly predicted as non-intrauterine growth restriction cases). Precision is defined as (correctly predicted as intrauterine growth restriction cases) / (correctly predicted as intrauterine growth restriction cases) + (incorrectly predicted as intrauterine growth restriction cases) [9].

## Results

Of 8,888 singleton deliveries, 205 were excluded because of congenital fetal abnormalities. We found 712 women with a recorded intrauterine growth restriction out of 8,888 deliveries, for a frequency of 8.19%.

**Figure 1:**
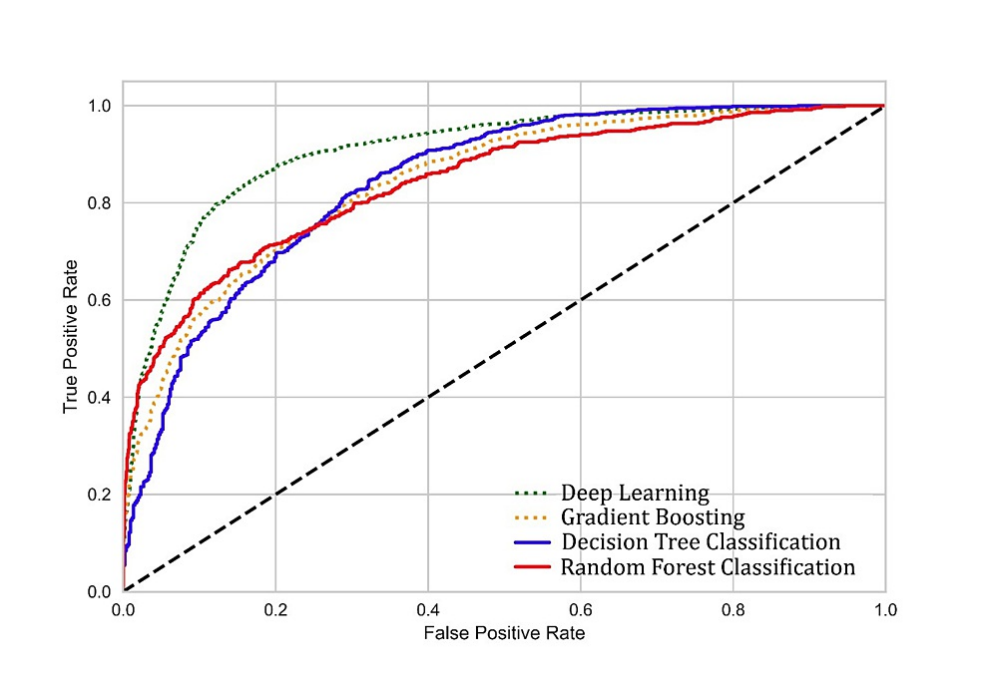
The AUROC curves of machine learning models AUROC: area under the receiver operating characteristic curve

The AUROC of each machine learning model is shown in Figure 1.

The AUROC for deep learning was 0.91 (0.85-0.97), showing the best performance compared to other models.

The other performance parameters, such as accuracy, precision, and sensitivity, of each algorithm are shown in Table 1.

**Table 1:** The performance of machine learning models AUROC: area under the receiver operating characteristic curve; CI: confidence interval

Algorithms	AUROC (95% CI)	Accuracy (95% CI)	Precision (95% CI)	Sensitivity (95% CI)
Deep Learning	0.91 (0.85-0.97)	0.88 (0.81-0.93)	0.72 (0.67-0.76)	0.52 (0.47-0.58)
Gradient Boosting	0.86 (0.83-0.9)	0.79 (0.73-0.82)	0.56 (0.54-0.58)	0.53 (0.49-0.59)
Decision Tree Classification	0.85 (0.79-0.92)	0.68 (0.65-0.71)	0.62 (0.59-0.66)	0.48 (0.43-0.52)
Random Forest Classification	0.84 (0.79-0.93)	0.61 (0.54-0.66)	0.53 (0.49-0.59)	0.42 (0.39-0.46)

Comparing the performance parameters of different machine algorithms showed that Deep Learning is the best model to predict intrauterine growth restriction.

**Figure 2:**
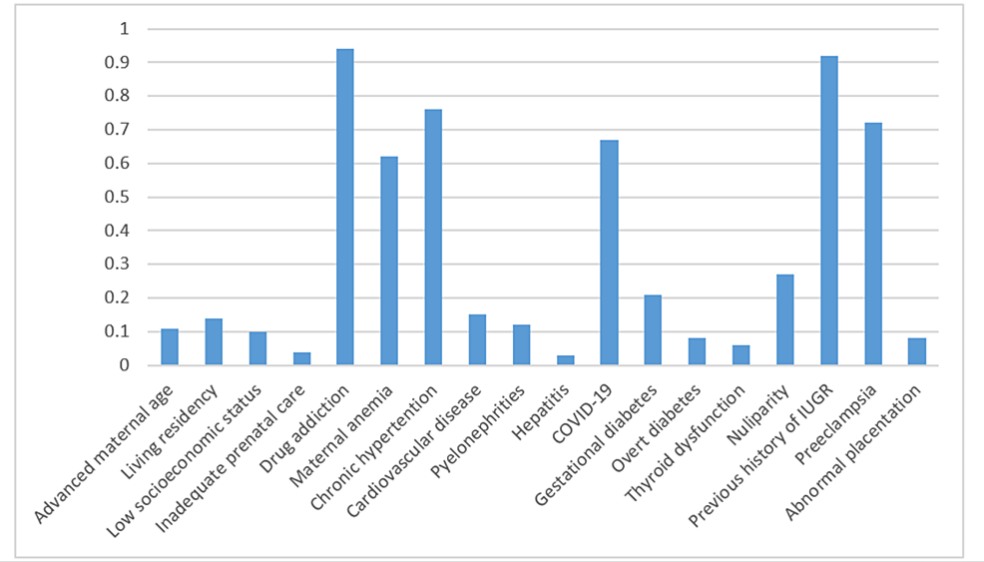
An analysis of the importance of variables in the Deep Learning algorithm IUGR: intrauterine growth restriction

Figure 2 presents an analysis of the importance of variables in the Deep Learning algorithm.

The importance of the variables revealed that drug addiction, previous history of intrauterine growth restriction, chronic hypertension, preeclampsia, maternal anemia, and COVID-19 were weighted factors in predicting intrauterine growth restriction.

## Discussion

Prediction of intrauterine growth restriction has the potential to alter the course of the disease, as these approaches will enable better follow-up to anticipate and diagnose the onset of the clinical condition, thereby preventing or mitigating the progress of fetal growth retardation. Thus, identifying maternal risk factors that predict intrauterine growth restriction in the early stages of pregnancy is essential. In this paper, we created a machine-learning model to detect intrauterine growth restriction. The model, in particular, can predict whether a pregnant woman will develop intrauterine growth restriction. As far as our knowledge is concerned, this is the first study in Iran that used a machine-learning approach to predict intrauterine growth restriction. Among different machine learning models, Deep Learning had a greater diagnostic performance in predicting intrauterine growth restriction with an AUROC of 0.91. The data from our study revealed that drug addiction, previous history of intrauterine growth restriction, chronic hypertension, preeclampsia, maternal anemia, and COVID-19 were weighted factors in predicting intrauterine growth restriction.

Substance abuse during pregnancy has been identified as a risk factor for negative birth outcomes, including intrauterine growth restriction [10]. According to our findings, opioid-dependent mothers were at higher risk of developing intrauterine growth restriction. Opioids circulating in the maternal blood can easily cross the placenta. Opioid drugs can alter the placental structure, transcriptome profile, and specific proteins like aromatase, decreasing uteroplacental blood flow and fetal growth [11].

Chronic hypertension and preeclampsia were among the predictors of intrauterine growth restriction. Hypertension damages the placenta, resulting in uteroplacental insufficiency. By 20 to 22 weeks of gestation, the pathogenic mechanism of the placenta seems to be a failure of trophoblastic invasion by maternal spiral arterioles. This failure results in luminal narrowing and medial degeneration, which reduce blood flow to the developing fetus. As a result, the fetus does not grow normally [12].

Anemia, as in our findings, has been proposed as a risk factor for intrauterine growth restriction, increasing the chances of adverse neonatal outcomes. Some biological mechanisms could link maternal anemia to intrauterine growth restriction. Low hemoglobin levels impair oxygen circulation in the body, resulting in an environment of oxidative stress or chronic hypoxia, which can lead to fetal growth restriction. Another possible mechanism for iron deficiency anemia is that iron deficiency stimulates the production of norepinephrine, which then stimulates the production of corticotropin-releasing hormone, potentially limiting fetal growth [13].

Women with a previous pregnancy history of intrauterine growth restriction were at higher risk of recurrent intrauterine growth restriction. Among our findings, COVID-19 was among the strongest predictors of intrauterine growth restriction. Initial studies on pregnant women have revealed that COVID-19 significantly increases the risk of intrauterine growth restriction [14,15]. Some studies have also shown maternal COVID-19 can affect the fetus's oxygen supply, resulting in placental insufficiency and intrauterine growth restriction [16].

The maternal body mass index is a significant modifiable risk predictor for intrauterine growth restriction [17]; however, this variable was among the missing data our study needed to analyze. This is a limitation of the study. Another limitation of our study was that the dataset size for the machine learning model was small. Even though we collected 8,683 cases, intrauterine growth restriction cases were only 712. The positive and negative cases in medical problems are usually uneven, which limits learning because the model cannot learn the pattern or features of positive cases from a small number of positive cases. Although internal validation was performed using cross-validation, external validation should also be performed to assess robustness.

## Conclusions

Deep Learning was found to be an accurate algorithm for predicting intrauterine growth restriction. The importance of the variables revealed that drug addiction, previous history of intrauterine growth restriction, chronic hypertension, preeclampsia, maternal anemia, and COVID-19 were weighted factors in predicting intrauterine growth restriction. However, appropriate algorithm improvement and refinement are necessary before implementation in daily clinical practice, and the importance of quality assessment and uniform diagnostic criteria should be further emphasized.
